# 

**Published:** 1883-04-20

**Authors:** 


### EDITORIALS AND MISCELLANEOUS.

### *EDITORIAL NOTICES.*

PROF. CARL VON HECKER, the great Obstetrican of Munich, died recently, in the fifty-sixth year of his age.

CHOLERA.—Something very like Cholera is prevailing to an alarming extent at Waterbury, Connecticut.

AMERICAN MEDICAL ASSOCIATION.—This great representative body of the medical profession in the United States, will meet the present year on the 5th of June, at Cleveland, Ohio.

OVERSIGHT.—The article in our March number entitled "*Look out for your Soft Catheter*," should have been credited to the *Peoria Medical Monthly.* We trust that excellent Journal will excuse the oversight.

COLDEN'S LIQUID BEEF.—Dr. W. Andrews, the gentlemanly agent of *Colden's Liquid Beef Tonic* (advertised in our Journal) gave us a friendly call and furnished us with a sample of that excellent nutritive tonic. It is neatly put up, agreeable to the taste, and truly a fine combination.

JOHN WYETH & BROTHER.—The gentlemanly agent of John Wyeth & Brother, Philadelphia, paid us a call and exhibited a number of beautiful samples from that excellent house. We have used Wyeth's preparations, and regard them as among the very best, the purest and neatest in the market.

THE STATE MEDICAL ASSOCIATION AT ATHENS.—There are indications of an unusually full attendance at the Medical Association of Georgia, which convenes at Athens on the 18th instant (April). It is to be hoped that all sections of the State will be represented; that impartial, kind and fraternal relations will prevail, and that the great interests of the profession will be advanced.

☞ SEE the card of Dr. JOHN G. WESTMORELAND under our *Special Notice* head. The Doctor, on account of age and declining health, finds himself unable to continue the arduous duties of out-door practice, but his patrons may still avail themselves of his long experience and established skill by consulting him in his office.

ANALYZING URINE.—Too little attention is paid by the profession to testing and analyzing urine, and unfortunately many practitioners are unacquainted with the proper methods or have not the facilities for making these tests, often indispensable to a correct diagnosis of disease. Such will do well to avail themselves of the services of Prof. J. H. LOGAN, whose card may be seen under our *Special Notice* head, and who is a Chemist of large experience and acknowledged ability.

BEAUTIFUL POCKET DRUG CASE.—Among the most unique and beautiful Pocket Drug Cases we have ever seen was one selected for the successful contestant for the prize in Physiology at the late commencement of the Southern Medical College. It was from the splendid drug house of McKesson & Robbins, New York. The vials were all filled with beautiful gelatine coated pills and granules. The size of the case, the plan of opening, the number of vials, the assortment of drugs, and the entire get up of the case, was the very acme of beauty, usefulness and convenience.

Established September 1, 1881. The Philadelphia Hospital for Skin Diseases, No. 923 Locust street, Philadelphia, Pennsylvania, is under the control of the following Board of Managers:

| | |
|---|---|
| Robert E. Rogers, M. D., | Hon. A. K. McClure, |
| William H. Pancoast, M. D., | William S. Jenney, M. D., |
| Rev. George F. Wiswell. D. D., | William B. Atkinson, M. D., |
| C. W. McKeehan, Esq., | Lawrence Wolff, M. D., |
| George W. Fairman, Esq., | George H. Brown, Esq. |

### *BOOKS AND PAMPHLETS RECEIVED.*

THE UNTOWARD EFFECTS OF DRUGS A PHARMACOLOGICAL AND CLINICAL MANUAL. By Dr. L. Lewin, Docent of Materia Medica and Public Health, in the University of Berlin. Second edition revised and enlarged. Translated by J. J. Mulheron, M. D., Professor of Principles of Materia Medica and Therapeutics in the Michigan Medical College, Detroit, Mich. The only English Translation having the author's endorsement. Detroit, Michigan, U. S. A. Geo. L. Davis, Medical Publisher, 1883.

The above is a work of 216 large octavo pages. Cloth, embossed sides and back, in black and gold. Price, $2 00. The work is exceedingly interesting and instructive in the information it imparts relative to the deviation of drugs from their ordinary action, or to occasional untoward results often not suspected by the practitiouer. It will well repay perusal.

Proposed Ordinance, Rules and Regulations for regulating the Plumbing, House Drainage, Registration and Licensing of Plumbers in the city of Philadelphia, as reported by the Committee of 21. Philadelphia, P. Blakiston, Son & Co., Medical and Scientific Publishers, 1012 Walnut street, 1883.

Suggestions regarding the local treatment of some of the commoner affections of the Ear, by Samuel Theobald, M. D., of Baltimore, Surgeon to the Baltimore Eye, Ear and Throat Charity Hospital; Ophthalmic and Aural Surgeon to St. Vincent's Hospital, Baltimore. Read before the Clinical Society of Maryland, November 17th, 1882.

Bromide of Ethyl, the most perfect anæsthetic for short, painful surgical operations, by Julian J. Chisolm, M. D., Professor of Eye and Ear Diseases in the University of Maryland, Surgeon-in-Charge of the Presbyterian Eye and Ear Charity Hospital; Ophthalmic Surgeon to the University Hospital, etc.

---

### *RECEIPTED.*

1882.—Drs. B T Phipps, James Wyly, Robert Duke, E King, Thos Norton, D D Tatum, E L Smith, Jas Phillips, R Eckhart.

1883.—Drs. J H Jennings, R J Talbert, B F Duke, W M Peacock, to March, '83; B R Bryant, to March, '83; A T Park, H Allison, W M Coleman, R F Pharr, 6 months; T C Davis, T E Morris, A W Irwin, James Ray, J M Benton.

